# Gait Type Analysis Using Dynamic Bayesian Networks

**DOI:** 10.3390/s18103329

**Published:** 2018-10-04

**Authors:** Patrick Kozlow, Noor Abid, Svetlana Yanushkevich

**Affiliations:** Department of Electrical and Computer Engineering, Schulich School of Engineering, University of Calgary, Calgary, AB T2N 1N4, Canada; pkozlow@ucalgary.ca (P.K.); noor.abid@ucalgary.ca (N.A.)

**Keywords:** gait, dynamic Bayesian network, Microsoft Kinect sensor, biometrics, human identification

## Abstract

This paper focuses on gait abnormality type identification—specifically, recognizing antalgic gait. Through experimentation, we demonstrate that detecting an individual’s gait type is a viable biometric that can be used along with other common biometrics for applications such as forensics. To classify gait, the gait data is represented by coordinates that reflect the body joint coordinates obtained using a Microsoft Kinect v2 system. Features such as cadence, stride length, and other various joint angles are extracted from the input data. Using approaches such as the dynamic Bayesian network, the obtained features are used to model as well as perform gait type classification. The proposed approach is compared with other classification techniques and experimental results reveal that it is capable of obtaining a 88.68% recognition rate. The results illustrate the potential of using a dynamic Bayesian network for gait abnormality classification.

## 1. Introduction

The need for more advanced human identification using biometric features has become a center of attention in fields such as security, surveillance, and forensics [1]. Among traditional biometric identifiers, such as facial features, human gait is especially interesting because research has shown that the way a person moves can be used for identification purposes [2]. An advantage of using the gait modality is that it can be linked with characteristics such as unobtrusiveness, effectiveness from a distance, and non-vulnerability, as it is difficult to continuously manipulate one’s own gait [2]. Additionally, the derivatives of gait biometrics can be used to address estimations of other secondary biometric characteristics such as age, gender, height, weight, or even emotional state [3].

There are several techniques used to observe an individual’s gait, including video data [4], physical markers [5], force plates [6], and electrodes attached to the skin or inserted into the muscle (EMG) [7]. However, most of these techniques are either too intrusive or not robust enough for applications related to surveillance or forensics because they involve too much physical interaction with the individual and/or introduce a large amount of error as a result of low quality source data.

As a solution to this problem, this paper examines using depth cameras such as the Microsoft Kinect which is capable of recording proximity and depth data in real time. In existing literature, approaches for gait analysis can be separated into two categories: model-free methods and model-based methods [8]. The first of the two approaches (model-free methods) is based on images that often consist of a human silhouette that is changing over time. Popular approaches within this category include gait energy images (GEIs) [8], frequency-domain features (FDFs) [9], and chrono-gait images [10]. These approaches have recently gained traction due to their simple yet effective properties. However, one common problem is that these approaches also tend to also be quite sensitive to viewpoints as well as scale which can lead to potential errors in noisy or uncontrolled environments [11].

On the other hand, model-based methods are based on the principle of tracking and modeling the body, which is represented by multiple parameters such as joint coordinates and their corresponding temporal relations. From the subsets, parameters such as joint coordinates as well as corresponding temporal relations can be plotted and used to derive patterns directly related to specific gait signatures. The output gait signature profiles can be used for identification and recognition depending on the application. Additionally, unlike model-free methods, model-based methods are generally view invariant as well as scale independent [11,12].

To classify individuals based on their gait patterns techniques such as hidden Markov models (HMM) [13], support vector machines (SVM) [14], and neural networks (NN) [15] are often utilized. Additionally, since gait is a sequence of temporal data, other types of classifiers like dynamic Bayesian networks (DBN) have also been used to assess the probability of an individual experiencing abnormal gait conditions [16].

In applications such as forensics, surveillance, or rehabilitation, results often rely on identifying evidence to describe an individual. Commonly used biometrics often assume the availability of a database or a watchlist. While databases of faces and fingerprints are commonly used in practice, no databases of “gait patterns” derived from video recordings are commonly available. However, the motivation of this paper comes from the knowledge that forensic databases may contain names and records of an individual, such as a mugshot, and/or other information pertaining to certain traits including scars, tattoos, approximate height, weight, or gait abnormality (e.g., a limp in the right leg).

**Figure 1:**
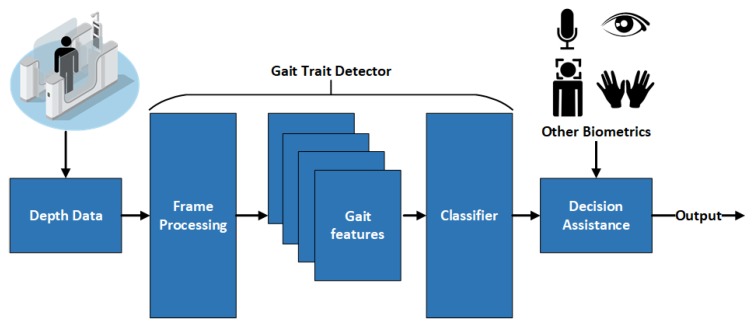
Architecture of the proposed framework and fusion for biometric screening enabled systems.

Thus, our study focuses on utilizing gait biometric characteristics, such as a limp, to improve screening applications using inexpensive sensors which can then be combined with other common biometrics to assist with verifying an individual’s identity. The main hypothesis of this paper is formulated as follows: human gait features derived using RGB-depth (RGB-D) cameras can be used to distinguish between different gait types such as normal versus abnormal (e.g., limping). To test this hypothesis, this paper proposes using the structure shown in Figure 1.

The novelty of the proposed approach includes the design of a DBN that is based on the information collected from RGB-Depth sensory data. This design is then used to generate decisions regarding an individual’s gait status. The proposed DBN is tested against other state-of-the art classifiers that also conduct gait type classification [17,18]. In [17], the authors attempted to classify Parkinson’s disease in subjects, while, in [18], the authors performed automatic recognition of altered gait using wearable inertial sensors for conditions such as Huntington’s disease and assess the risk of a potential stroke.

The reason DBN was chosen over other classifiers is because of its unique properties, such as causality and ability to model temporal sequences such as gait. For example, in [16], the authors demonstrated the usefulness of applying probabilistic models such as a DBN to assess gait. Additionally, the authors in [13] presented a two-level DBN layered time series model (LTSM) to solve and improve on other gait classification methods such as HMM and dynamic texture models (DTM). The results from [13,16] further demonstrate the validity of the proposed model and its potential for other fields such as biological sequence analysis and activity recognition.

Nonetheless, a DBN is just one of many methods that can be used to model temporal relations. Other state-of-the art modeling techniques such as the generative probabilistic model with Allen’s interval-based relations have been created to effectively model complex actions using various combinations of atomic actions [19]. The atomic actions are referred to as primitive events inferred from sensor data which can no longer be decomposed into a lower-level semantic form [20]. These actions represent an intermediate form between low-level raw sensor data and high-level complex activities. However, it is assumed that the atomic actions are already recognized and labeled in advance [19]. In this paper, the features used for gait detection are automatically labeled by the proposed system when fed raw sensor data from the Kinect camera. Acquiring feature data in this format eliminates the need for assuming feature labels and provides insight into building a system based off of real data.

The rest of this paper is organized as follows. A description of our method is presented in Section 2. Experimental results are described in Section 3, and a discussion of the results is presented in Section 4. The concluding remarks and future plans are presented in Section 5.

## 2. Materials and Methods

### 2.1. Definitions

A person’s gait is a coordinated motion that is repeated over a time series. By engaging muscles such as the quadriceps and hamstrings, an individual is able to propel themselves forward. One unique property of gait is that it is cyclical and symmetric unless an abnormality is present. The normal progression for gait begins when one foot becomes load bearing as the opposite foot lifts off the ground. In this moment, various joints and muscles make adjustments to keep the center of gravity located near the torso stable. As the unburdened foot swings forward past the load bearing foot, it begins to lower and the process repeats for the opposite side of the body.

The most common way to measure this process is by using a gait cycle. A gait cycle is a measurement that is a function of time and is commonly separated into percentiles. The gait cycle begins when one foot becomes load bearing and the opposite foot begins to lift off. The end point can be marked when the individual reaches the same position as the start of the gait cycle after the opposite foot has completed the swing phase and the loading phase.

**Figure 2:**
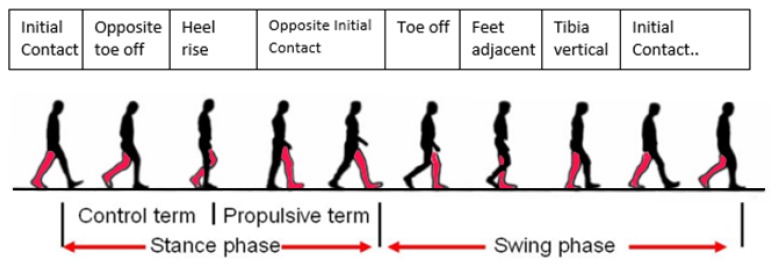
The seven sequential phases of the gait cycle.

To illustrate the gait cycle more clearly it is commonly modeled as a sequence of seven phases as shown in Figure 2 [21]. For additional clarity, the foot that begins the gait cycle is addressed as the primary foot and the opposite is labeled as the secondary foot. More details regarding the gait cycle can be found in [22].

The above can be used to describe ideal gait; however, in the real world, many individuals do not have this type of gait. As stated above, when an abnormality is present, the cyclical or symmetric properties of gait are often affected [23]. Investigating this relation is one of the underlying motivations of this paper because the abnormalities or characteristics of one’s gait can be as supporting biometric evidence in addition to other biometric modalities such as fingerprints, or iris patterns to establish congruent identity.

### 2.2. Sensor: Kinect v2 by Microsoft

The sensor we used for this investigation was the Kinect v2 RGB-Depth camera. Capable of acquiring RGB data, NIR data, and depth data at 30 frames per second, the Kinect v2 is a major improvement to its predecessor the Kinect v1, as shown in Table 1. It has been used in a wide variety of experiments related to object recognition, 3D reconstruction, and motion tracking [24]. Exploring gait via model-based approaches is reported in [12,17,25,26,27,28]. This is because the Kinect is capable of collecting depth information as well as deriving skeletal information in real time.

One of the main reasons for choosing the Kinect v2 is because of the improvement of the depth tracking technology compared to the previous version. Unlike the Kinect v1, which used structured light to obtain depth data, the Kinect v2 utilizes Time of Flight (ToF) technology which allows for higher resolution and lower image degradation from lighting and environmental noise [29]. The ToF technology that the Kinect v2 uses is based on emitting IR beams that reflect off of the target object. The time traveled by the emitted beams to the object and back is measured and used to derive the distance from the camera. In addition, the obtained distance information can also be used to form a point cloud, which can be used to further derive a skeleton model.

**Figure 3:**
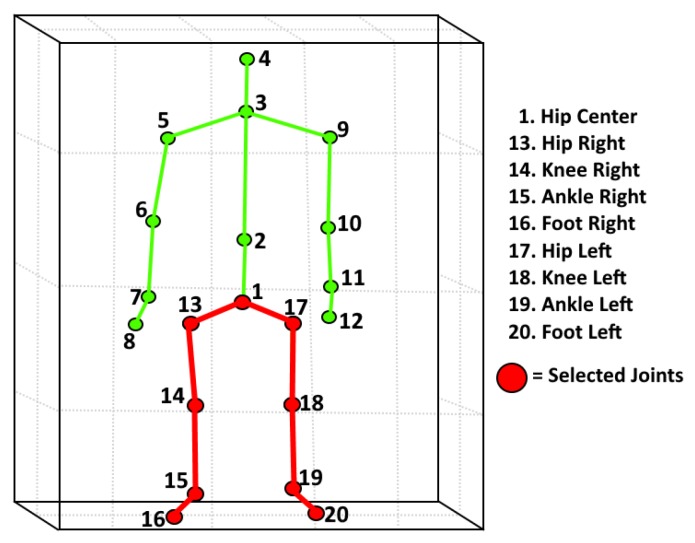
Lower body joints and connections selected for gait feature extraction as indicated by the red color.

An essential characteristic of the Kinect system is its embedded capability for collecting depth information as well as the ability to derive skeletal information in real time. The skeletal model used for this paper was derived using the NUI Skeleton library which is a part of the Kinect Software Development Kit (SDK) released by Microsoft. Figure 3 illustrates the skeleton model generated using the sensor and SDK. For the purpose of this paper, it was decided to use only the lower body joints of the skeleton for gait analysis because these joints inherently experience more change when an individual’s gait begins to deteriorate, as shown in [30].

A review of research on accuracy of the Kinect v2 is reported in [31]; seven studies compared the Kinect to the Vicon system as a gold standard, and two studies compared it to the Optotrak Certus system. The agreement between the Kinect and the selected gold standard was assessed using Bland-Altman 95% bias as well as limits of agreement (LoA), Pearson’s correlation coefficients, ICC, or concordance correlation coefficients. It confirmed that step width, step length, and stride length all demonstrated excellent agreement. Additionally, gait speed, step time, and stride time also all showed moderate to excellent agreement. However, in Mentiplay et al. [32] the authors found that pelvis displacement and ankle flexion did not share much agreement when compared to the gold standard. These findings will be used to validate our feature vector used for classification.

It should be noted that the Microsoft Kinect camera was officially discontinued in 2017. However, the work presented in this paper remains transferable to any depth camera currently available due to nature of the developed framework. In other words, current cameras such as the Intel Realsense d435, can be used to continue this research by performing tasks such as skeleton extraction and 3D modeling in real time when configured with an applicable software development kit (SDK) or open source library (OpenCV).

### 2.3. Experiments and Data Collection

The method proposed in this research can be separated into three phases: data collection, pre-processing, and classification. The data collection portion was done using a custom C++ program, which was then used as an input into a script made with MATLAB 2016b to conduct the pre-processing and feature extraction. The classification portion was done using Hugin (Hugin Experts Aalborg, Denmark) [33], a sophisticated software package capable of modeling and testing various types of causal networks, including DBNs.

#### 2.3.1. Experimental Setup

**Figure 4:**
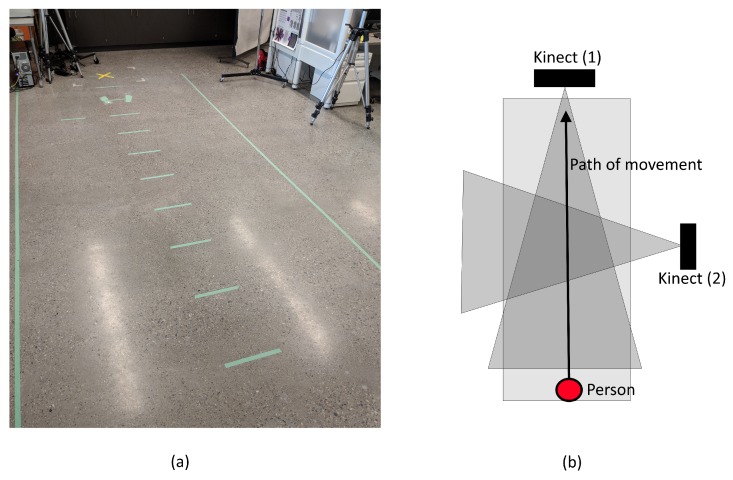
Diagram of setup used for gait data collection: (**a**) the actual setup; and (**b**) a view from above.

For this study, the publicly available data were limited. Therefore, it was necessary to collect a local dataset that could be used for testing. To record the necessary gait sequences, two Kinect cameras were set up; one at the front (frontal) and one at the side (perpendicular) with respect to the subject’s path. This setup is illustrated in Figure 4. The tilts and positions of the cameras were orientated in such a way that the whole body of the subject would be visible during recording. In regards to the environment itself, we created a track that measured 4.5 m in length and 1 m in width separated by 0.5 m increments. The reason for the proposed setup is two-fold. First, since the depth sensor in the Kinect has a maximum range of 4 m, it will not detect the subject until they have begun walking for at least 0.5 m. This allows the camera to record the subject’s natural moving gait cycle, as opposed to their gait cycle from rest. Second, the 0.5 m increments allow us to verify the depth readings with the true position obtained from the synchronized RGB video.

#### 2.3.2. Data Collection

In our experiment, 28 healthy subjects from the University of Calgary were asked to perform the experiment which consisted of six sequences in the following order: two normal walking, two with a left limp imitation, and two with a right limp imitation. Details for each subject are shown in Table 2. Additionally, we supplemented our data by combining it with the UPCV dataset [26,34], which consisted of 30 people providing five recordings each, resulting in 150 additional normal gait samples. After combining the datasets, the total number of samples used for this investigation was 318. For experimentation, the combined dataset will be partitioned into two subsets: Dataset A, which consists of locally collected data (Ucalgary), and Dataset B, which represents the entirety of the local and external datasets (Ucalgary and UPCV) combined. Details regarding the subsets can be seen in Table 3.

For these experiments, we selected a gait type (antalgic, also known as “limping”) that is easy to reproduce in order to prove that it is feasible to distinguish different gait types via non-contact methods. Another reason we chose to experiment on a limp is because it would give us data that could be comparable to other known results such as [17,18,35].

### 2.4. Data Processing

To process the data, spatiotemporal data were first retrieved by the Kinect v2 sensor and stored in an N×M matrix where *N* represents the number of features and *M* corresponds to the number of frames per sequence. For this application, N=21 and M=120, where *N* represents the Cartesian coordinates (X,Y,Z) of seven features being stored (3×7=21) for a total of 120 frames. These matrices were output by the C++ application in CSV format and used in Matlab 2016b and Hugin Expert for further analysis. To create the proposed DBN, the following processing were required: feature selection, event labeling, and network design.

#### 2.4.1. Feature Selection and Relations

The features that we use to create the DBN were selected based on expert analysis and results obtained in [16]. The features shown in Table 4 represent a subset of features selected from a list containing 31 unique features ranging from ambulation time (s) to toe in/out angles (${}^{\circ }$) [36]. To validate the selected methods, similarities and differences in selection proposed in [16] were compared with methodology presented in [37] and the proposed features selected for this study. We found that the features selected to train and test the proposed network shared many of the same features as used in [16] and [37]. In this paper, abbreviations have been changed for clarity. For the system proposed in this study, the following features were selected: Cadence (CAD), left ankle joint angle (LJA), left knee joint angle (LJK), right ankle joint angle (RJA), right knee joint angle (RJK), left stride length (LSL), and right stride length (RSL). Compared to the features in [16,36], four new features were introduced. These features are the joint angles for the knees and ankles that correspond to an individual’s gait during locomotion. To compute the joint angle from the coordinates at any given frame, Equations (1)–(3) were used.

(1) $$\begin{array}{c} \Delta \left(p_{1},p_{r}\right)=\sqrt{{\left(x_{1}-x_{r}\right)}^{2}+{\left(y_{1}-y_{r}\right)}^{2}+{\left(z_{1}-z_{r}\right)}^{2}} \end{array}$$

(2) $$\begin{array}{c} \Delta \left(p_{2},p_{r}\right)=\sqrt{{\left(x_{2}-x_{r}\right)}^{2}+{\left(y_{2}-y_{r}\right)}^{2}+{\left(z_{2}-z_{r}\right)}^{2}} \end{array}$$

(3) $$\begin{array}{c} \Theta \left(p_{1},p_{2},p_{r}\right)=\frac{\cos^{-1}\vec{P_{1}}\cdot \vec{P_{2}}}{\Delta \left(p_{1},p_{r}\right)\Delta \left(p_{2},p_{r}\right)} \end{array}$$

where finding the angles between two joints $p_{1}$ and $p_{2}$ can be defined as the angle formed by $p_{1}$ and $p_{2}$ with respect to a reference joint $p_{r}$. The reference point can be any joint that connects two different joints together. For example, the ankle can be used as a reference point to establish the connection between the foot and the knee. The reason for choosing these specific joint angles is based on findings in [35] where the authors successfully utilized the angles corresponding to the knees to differentiate between two different gait types.

After selecting the features shown in Table 4, the next step was to establish the connections between the features when represented in a model or node form. In our proposed decision network, we wanted to use features that were closely correlated (R≈|1|) for a given set of observations $\left(x_{n},y_{n}\right)$ as well as their respective total scores $S_{x},S_{y}$, where α represents an experimentally determined coefficient that takes a value between −1 and 1 −1≤R≤1, with 1 or −1 indicating perfect positive or negative correlation respectively as shown by Equation (4):

(4) $$R=\frac{1}{n-1}\sum \left(\frac{x-\bar{x}}{S_{x}}\right)\left(\frac{y-\bar{y}}{S_{y}}\right)$$

**Figure 5:**
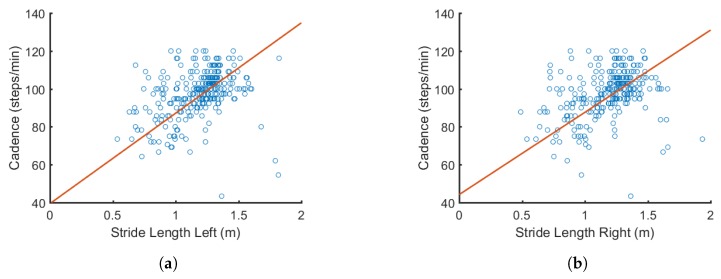
Example of how cadence is correlated with: (**a**) left stride length (R=0.6941); and (**b**) right stride length (R=0.6529). Correlations are later used to form the connections in the DBN.

Figure 5 illustrates how some features such as cadence and stride length are related to each other. From resulting calculations, we found that the *p* value was much less than 0.001, indicating that the correlation coefficient *R* is statistically sufficient. A similar approach was used to determine the correlation between the other connecting nodes in the DBN. This is a critical step in the pre-processing stage because it determines the connectivity between the nodes selected to be used in the proposed network.

#### 2.4.2. Event Labeling

**Figure 6:**
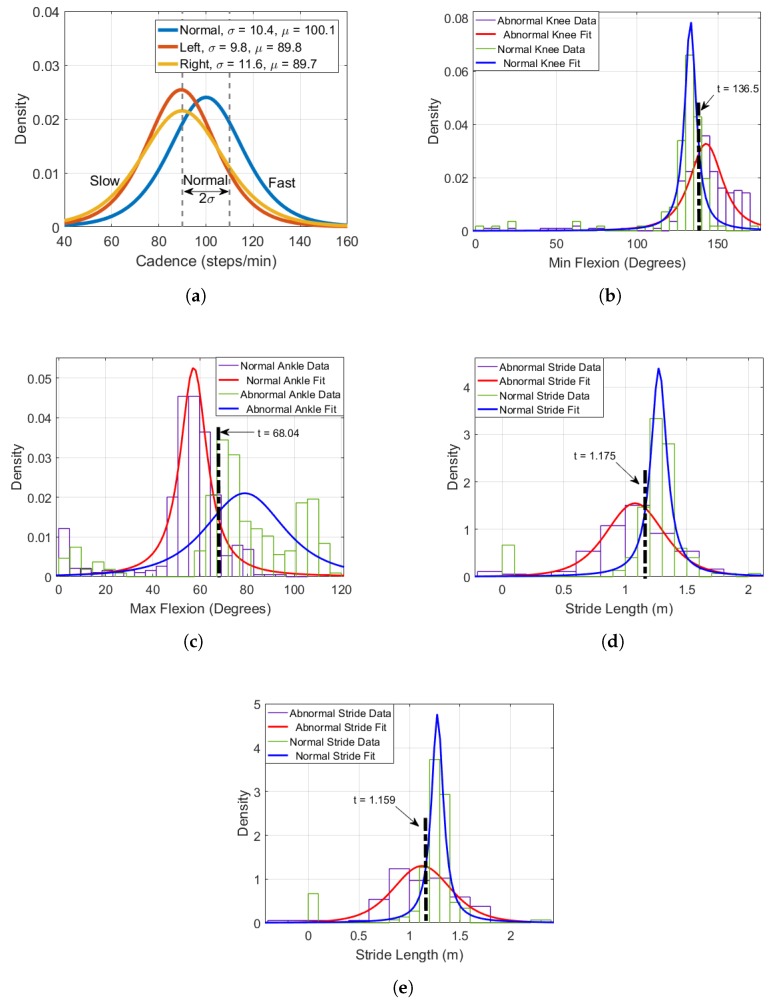
PDFs of features such as: (**a**) cadence; (**b**) minimum knee flexion; (**c**) maximum ankle flexion; (**d**) left stride length; and (**e**) right stride length, selected for the proposed DBN.

One challenge about using a DBN in this application is that the labels associated with each node are qualitative and descriptive as opposed to a set of integer values. Likewise, the input data must also be in this format. To reformat the input data from a numerical sequence to a matrix of labels, some statistical processing was necessary. An example illustrating the probability density functions (PDFs) for features such as cadence (CAD) can be seen in Figure 6a.

Here, the figure illustrates how various outcomes are assigned for events such as CAD. To assign outcomes for continuous probability distributions of features, the mean and standard deviation is first computed. Next, the literature was examined to verify the assignment of the outcomes. For example, for CAD (one of the selected features), the work in this thesis examined the literature [38,39,40] and found that normal cadence for adults was defined to exist in the range between 90 and 120 steps per minute. The results in Figure 6a reflect similar observations with the mean μ = 100.1 (steps/min) as well as a standard deviation of σ = 10.4. From these observations, the assumption was made that any measured cadence within one standard deviation to the left or right side of the normal curve mean is considered normal, this corresponds to approximately 65% of the total cadence data. To label abnormal gait which contains either slow or fast, the following range was used:

(5) Slow<90andFast>110

To summarize, the cut off threshold proposed can be represented as follows:

μ(normal)+1σ

This equation is used to minimize bias for future tests done on the UPCV and UCalgary datasets. PDFs for the other features reported are also examined and shown in Figure 6b–e, where *t* is used to indicate the threshold value. The value *t* represents the experimentally defined threshold that is used to categorize an individual’s gait features. One problem of using this approach is that it introduces some error into the labeling process due to the uncertainty for data located at the threshold of the distributions; nonetheless, this step is necessary to format the data appropriately.

#### 2.4.3. Dynamic Bayesian Network Design

Dynamic Bayesian Networks (DBNs) are a useful way to represent temporal processes such as gait. This is because they are comprised of two parts: a static Bayesian Network (BN) that can be used to compute the probabilities of an event occurring, and a transitional BN, which models the temporal relations of selected variables.

**Figure 7:**
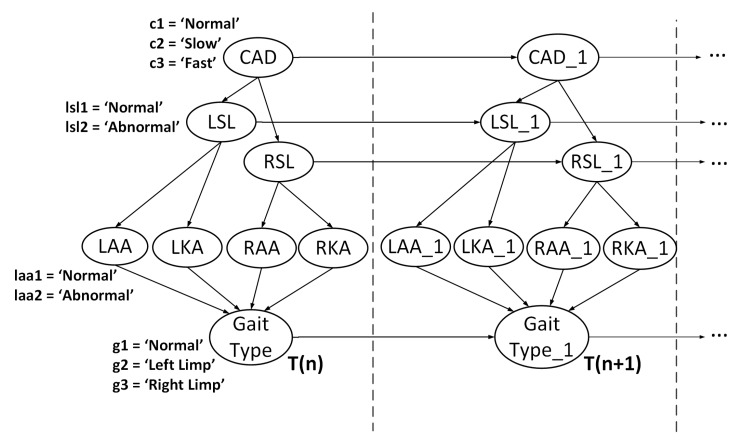
Proposed DBN for gait type recognition.

The DBN is created using the features and labels defined in Table 4, where the nodes in the network correspond to the proposed features and the connections between the nodes are defined by using the correlation values computed in Section 2.4.1. To assign probabilities for each node, conditional probability tables (CPTs) were created based on the PDF distributions illustrated in Figure 6. The initial CPTs chosen for the proposed approach are shown in Table 5. After selecting and labeling the nodes, the resulting DBN is illustrated in Figure 7, where the number of time slices corresponds to the number of gait cycles recorded.

To train and test the DBN, this study used Hugin (Hugin Experts Aalborg, Denmark) [33], a sophisticated software package capable of modeling and testing various types of causal networks. During training, we utilized sequential updating [41], also known as adaptation or sequential learning to update the CPTs in the network. The adaptation algorithm functions based on the notion of experience, which is a quantitative memory that is based on both expert judgment and past cases. Experience can be represented as a set of “counts” $a_{0},a_{1},\ldots ,a_{(}n-1)$, where *n* is the number of configurations of the parent nodes. Here, $a_{i}$ represents number of times the parent nodes have been observed in the *i*th configuration. The experience “counts” are stored in a table often referred to as an experience table. When initialized, an experience table is set to zero. To perform adaptation, positive values must be stored in the table [42].

Experience consists of a probability distribution P(Θ) over a parameter space Θ, and contains a core which is a joint distribution abbreviated as P(V|Θ). The core is used to express the relationships between the nodes in the DBN. Experience and the core are linked via dissemination which is used to produce a marginal distribution P(V) as follows:

(6) P(V)=∫P(V|Θ)P(Θ)dΘ

P(V) can be used to evaluate the event *E*. The core, or node, that is being updated with some data *E* is updated based on the posterior probability distribution P(Θ|E).

**Figure 8:**
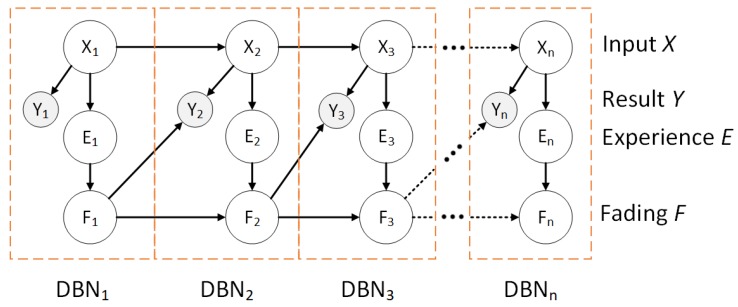
A depiction of experience and fading for *n* time slices in a DBN.

One disadvantage of using experience over a sequence of cases is that it creates a bias which assumes that all experience is relevant. Intuitively, past experiences should be gradually eliminated in order to give more recent experiences more impact on the outcome. Fading is a method proposed in [43] that does exactly this; making nodes ignore things they have learned a long time ago. To apply fading, the experience count $a_{i}$ is discounted by a fading factor $q_{i}$ which is a positive real number that is less than but typically close to 1. An example of fading can be represented by the following equation:

(7) $$P_{i}=a_{i}\left(1-p_{i}+p_{i}q_{i}\right)$$

where the *i*th parent given the propagated evidence $P_{i}$ is computed before adaptation takes place. Similar to experience, the fading variable for a node is stored in a fading table that has a range of [0, 1], where 1 indicates no fading (all experience is stored) and 0 indicates maximum fading (all experience is ignored). Figure 8 illustrates a high level model of this approach. For each time slice, except for the initial slice (n=0), the network stores the input information *X* as a form of experience *E* which is discounted by a fading factor *F* which is then propagated to the future time slice to compute the result *Y*. Table 6 illustrates how the CPTs adapt after training the DBN.

According to Lauritzen and Nilsson [44], adaptation is especially useful in this application because the modeled domain is drifting over time in addition to being initialized using simple values. The proposed model was evaluated using leave-one-out cross validation (LOOCV). To perform LOOCV, the observations are left out one at a time at each time segment in the DBN. The process is repeated for all the observations, and the predictive power is evaluated as the average of all instances. The purpose of using this validation technique is to estimate the average performance of the system with a relatively low sample set (≤1000) [16].

## 3. Results

For this study, two experiments were conducted based on the partitioned data defined in Table 3. For the first experiment, the abnormal classes (left limp and right limp) were combined into one class (abnormal). This allowed for easy comparison with the other classifiers tested. To test the network, we used the Hugin Expert Lite model which allowed for a maximum of three time slices. Unfortunately, because of software limitations, we were not able to train the DBN on a longer time series (T≥3). Therefore, some accuracy might have been sacrificed for the sake of simplicity. However, overall results remain promising. To calculate Correct Classification Rate (CCR), sensitivity, and specificity the following formulas were used:

(8) $$\begin{array}{c} CCR=\frac{TP+TN}{TP+TN+FP+FN} \end{array}$$

(9) $$\begin{array}{c} Sensitivity=\frac{TP}{TP+FN}*100 \end{array}$$

(10) $$\begin{array}{c} Specificity=\frac{TN}{TN+FP}*100 \end{array}$$

where TP is the number of true positives (correctly predicting the gait type of an individual as normal); FN is the number of false negatives (incorrectly predicting an abnormal gait type); TN is the number of true negatives (correctly predicting an abnormality (left limp or right limp)); and FP is the number of false positives (abnormal gait types being predicted as normal).

The corresponding performance evaluation results are presented in Table 7 and Table 8. In addition to DBN, four other classifiers were used for comparison: K-nearest-neighbors (KNN), support vector machines (SVM), naïve Bayes (NB), and linear discriminant analysis (LDA). All of the mentioned classifiers were trained and tested on Dataset A in Table 3 which consisted on locally collected data using five-fold cross validation. In Table 7, the best results were observed with the proposed DBN, while the worst are seen with LDA. A comparable CCR was also obtained with a NB classifier. However, the CCR for the NB was still 10% lower than the DBN. As seen from the results, the DBN is effective at distinguishing normal from abnormal gait for Dataset A.

For the second experiment, the dataset was expanded. It included local as well as external data from two different datasets. The data selected for the second experiment consisted of local data used in the previous experiment combined with UPCV Kinect data from the University of Patras Computer Vision Group [26,34]. The purpose of this experiment was to observe how the selected classifiers CCR changed when additional data was introduced into the training and testing set. In Table 8, it can be seen that the DBN once again achieved the highest CCR for binary classification. However, the CCR of all the other classification techniques also increased, with the most improvement observed in the SVM classifier (+19.32%).

**Figure 9:**
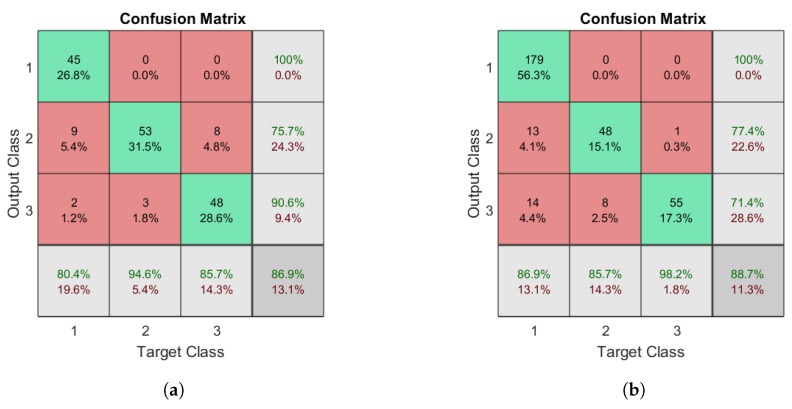
Confusion matrices for multi-class recognition using a DBN: (**a**) the matrix for Dataset A; and (**b**) the matrix for Dataset B. The target classes are as follows: 1, normal; 2, left limp; and 3, right limp.

In the second experiment, the abnormal class was in the DBN was separated so that it contained two different output classes for gait type: left limp, and right limp. To determine how efficient the DBN is at performing multi-class classification, the network described in Figure 7 was tested on a newly labeled dataset that contained normal, left limp, and right limp gait types. The resulting confusion matrices for Datasets A and B are illustrated in Figure 9a,b, respectively. The corresponding precision and recall for the classes ranged 71–100% and 86–98%, respectively, depending on the data used for testing and training.

To compare the results with the outcomes of other authors, the binary classification results of the DBN were used. However, the strength of the proposed network is that is robust to multi-class recognition. Results were compared with literature where the authors conducted similar experiments using contact and non-contact systems to detect specific gait abnormalities. At the time of conducting this study, the following studies were used for comparison [17,18,35].

## 4. Discussion

The obtained results from the DBN were 88.68% CCR for multi-class classification (normal, left limp, right limp). These results are promising, however, to validate the results, they were compared against three similar studies.

The first article selected for comparison is by Kozlow et al. [35]. The authors conducted gait detection via binary classification using information collected from the Kinect v2. In their study, the authors used two features to form the feature vector: minimum ankle flexion and maximum ankle flexion. From their experiments, the authors found that using KNN resulted in a 88.54% CCR. However, after repeating the experiments using the selected dataset, the CCR dropped to 83.45% when trained/tested using 21 features. When compared with the DBN proposed in this study, we observed that the DBN achieved a higher CCR for multi-class classification (88.68%).

The first article selected for comparison is by Prochzka et al. [17] who examined using depth data from the Microsoft Kinect to perform Bayesian classification for Parkinson’s disease. The results from their study yielded good results ranging 90.2–94.1% CCR using features such as stride length and speed. However, to obtain the maximum CCR (94.1%), the researchers needed to include age into the feature vector. This is questionable, as age is not always known and needs to be acquired via manual questioning or approximated (which can introduce error into the system). The DBN proposed for this study results in a slightly lower CCR than observed in [17]. However, the output class labels for antalgic gait are inherently more interrelated than the classes associated with Parkinson’s. They are also calculated using data that can be directly acquired using only the Kinect v2.

The final article [18] contains information about an experiment conducted that attempts to perform automatic recognition of altered gait using wearable inertial sensors. In [18], Mannini et al. examined 54 individuals with various known conditions such as being prone to stroke, Huntington’s disease and Parkinson’s disease, as well as healthy elderly individuals. Using classification methods such as NB, SVM, and logistic regression (LR), the authors attempted to maximize the discrimination capabilities of each classifier to correctly classify the data. Results from this study found that LR yielded the highest CCR (89.2%) when using inertial measurement units mounted on the subject’s shanks and over the lumbar spine. Comparing the proposed DBN to this literature shows that a similar CCR is obtainable when using exclusively non-contact methods, which are more favorable in real life applications such as border control. A comparison of the proposed method and that in [18] is shown in Table 9.

We compared the overall accuracy of our proposed network to the ones reported in [17,18,35] in Table 9. In the table, we can observe that the proposed method demonstrated a very good performance compared to the other methods. However, the DBN underperformed in comparison to the NB classifier presented in [17]. This could be because the results presented were based on a relatively small dataset (51 samples) which was not publicly available, as well as the classes created were relatively easy to distinguish due to large separation between the classes. What makes our result unique as well as robust is that it was tested on over 300 samples from two different datasets, as well as on closely interrelated classes.

## 5. Conclusions

This study contributes to the development of future biometric-enabled technologies where automated gait analysis may be applied. Here, soft biometrics such as gait type are used to provide a more robust decision making process regarding individual’s identity (expressed in terms of distinguishing feature such as a limp) or gait deterioration analysis in non-invasive, remote sensor based analysis of walking patterns. The classification of gait type proposed in our work is based on a probabilistic inference process, via DBN. This approach is expandable to overall identity inference process that utilizes statistics collected using available or recorded biometrics of a individual’s face, fingerprint, and iris. It can be paired with other soft biometrics such as age, height, or other traits to assess identity, or used for analysis of gait for rehabilitation process monitoring and other healthcare applications.

The proposed gait feature analyzer is a framework that is able to detect gait abnormalities in a non-invasive manner using RGB-depth cameras. We examined the potential application for this framework by diagnosing various types of antalgic gait. Using a Kinect 2.0 sensor, we collected 168 samples which consisted of equal portions of normal gait, and simulated antalgic gait (for the left and right side). By using frame by frame analysis of the individual’s 3D skeleton, we were able to model the individual’s gait cycle as a function of frames. The gait cycle was then utilized to derive critical features for identifying one’s gait. Features such as cadence, stride length, and various knee joint as well as ankle joint parameters were selected. These selected features were used for testing a novel DBN that will output one’s gait type in a semantic form.

From experimental results, we found that the proposed DBN was effective for multi-class decisions (different types of gait) yielding an overall CCR of 88.68%. When compared to other state-of-the art methods, the proposed solution was comparable with all but one result which yielded 94.1% that also utilized Bayesian classification techniques (NB). There are many possible reasons the proposed network was unable to reach this level of accuracy, which include: not enough time sequences for an individual, or that the dataset we attempted to classify had strong interdependent properties, as well as having a limited amount of features that frequently contained overlapping PDFs.

In future work, we plan to address several limitations that currently exist. First, the database we collected that contains abnormal gait types is still relatively small and uniform. We plan to continue to improve the size and diversity of the database, including additional classes to improve the robustness of the framework. In addition, we will also continue to add more distinctive features that will be used to identify an individual’s gait type with higher accuracy. Lastly, we plan to explore more complex and powerful networks, including deep neural networks, provided enough input data.

The proposed gait feature analyzer is yet to be integrated with biometric-enabled screening architecture (Figure 1). For example, forensic databases or watchlists often contain face or fingerprint data and a textual records, such as information about distinctive gait feature or abnormalities. Automated identification of these "soft" biometrics in the screening process (such as a person walking through airport border control) will allow to improve performance of the identification system, or assist in decision making based on multiple trait acquired during the screening process or on the video

## Figures and Tables

**Table 1:** Specifications of the two Kinect cameras.

Feature	Microsoft Kinect v1	Microsoft Kinect v2
Measurement Method	Structured Light	Time of Flight
Color Camera at 30 fps	640 × 480	1920 × 1080
Depth Camera	320 × 240	512 × 424
Skeleton Joints Defined	20 joints	26 joints
Number of Models Tracked	2	6
Minimum Latency (ms)	102	20–60

**Table 2:** Participant details for local dataset.

Subject No.	Sex	Age	Height (cm)	Subject No.	Sex	Age	Height (cm)
1	F	47	167	15	M	22	185
2	M	26	182	16	M	22	178
3	F	27	177	17	M	27	177
4	M	24	191	18	M	23	165
5	F	28	168	19	M	22	162
6	M	22	190	20	M	23	185
7	M	24	187	21	M	23	180
8	F	28	157	22	M	22	181
9	F	35	162	23	M	22	184
10	M	25	170	24	M	23	163
11	F	22	166	25	M	22	168
12	F	22	173	26	M	22	198
13	M	22	183	27	M	23	186
14	F	26	160	28	F	26	153
Mean ± STD						25 ± 5.2	174.9 ± 11.6

**Table 3:** Dataset partitions used for this study.

	Dataset A	Dataset B
Total Gait Sequences	168	313
Normal Gait Sequences	56	201
Abnormal Gait Sequences	112	112

**Table 4:** Comparing selected features.

Expert Selection [36]	FSS [16]	Proposed Network
Cadence (steps/min) (CAD)	Right extremity ratio (RPED)	Cadence (steps/min) (CAD)
Left step length (cm) (LPI)	Right step time (s) (TPD)	Left ankle joint angle (${}^{\circ }$) (LJA)
Right step length (cm) (LPD)	Left double support (%) (SDI)	Left knee joint angle (${}^{\circ }$) (LJK)
Base support left step (cm) (BSI)	Base support left step (cm) (BSI)	Right ankle joint angle (${}^{\circ }$) (RJA)
Base support right step (cm)(BSD)	Base support right step (cm) (BSD)	Right knee joint angle (${}^{\circ }$) (RJK)
Left stride length (cm) (LSL)	Right toe in/out angle (${}^{\circ }$) (TIOD)	Left stride length (m) (LSL)
Right stride length (cm) (RSL)	Left toe in/out angle (${}^{\circ }$) (TIOI)	Right stride length (m) (RSL)

**Table:** (**A**) Initial CPT for Cadence

CAD${}_{T-1}$	Fast	Slow	Normal
Fast	0.8	0.05	0.05
Normal	0.05	0.8	0.05
Slow	0.15	0.15	0.9

**Table:** (**B**) Initial CPT for Left Stride Length

LSL${}_{T-1}$		Normal			Abnormal	
**CAD**	**Fast**	**Slow**	**Normal**	**Fast**	**Slow**	**Normal**
Normal	0.9	0.9	0.95	0.1	0.1	0.25
Abnormal	0.1	0.1	0.05	0.9	0.9	0.75

**Table:** (**C**) Initial CPT for Right Stride Length

RSL${}_{T-1}$		Normal			Abnormal	
**CAD**	**Fast**	**Slow**	**Normal**	**Fast**	**Slow**	**Normal**
Normal	0.9	0.9	0.95	0.1	0.1	0.25
Abnormal	0.1	0.1	0.05	0.9	0.9	0.75

**Table:** (**D**) Initial CPT for Left Ankle Joint Angle

LSL	Normal	Abnormal
Normal	0.9	0.1
Abnormal	0.1	0.9

**Table:** (**E**) Initial CPT for Left Knee Joint Angle

LSL	Normal	Abnormal
Normal	0.75	0.1
Abnormal	0.25	0.9

**Table:** (**F**) Initial CPT for Right Ankle Joint Angle

RSL	Normal	Abnormal
Normal	0.9	0.1
Abnormal	0.1	0.9

**Table:** (**G**) Initial CPT for Right Knee Joint Angle

RSL	Normal	Abnormal
Normal	0.75	0.1
Abnormal	0.25	0.9

**Table:** (**A**) Trained CPT for Cadence

CAD${}_{T-1}$	Fast	Slow	Normal
Fast	0.186	0.057	0.099
Normal	0.226	0.558	0.359
Slow	0.589	0.385	0.542

**Table:** (**B**) Trained CPT for Left Stride Length

LSL${}_{T-1}$		Normal			Abnormal	
**CAD**	**Fast**	**Slow**	**Normal**	**Fast**	**Slow**	**Normal**
Normal	0.545	0.384	0.579	0.299	0.224	0.504
Abnormal	0.455	0.616	0.421	0.701	0.776	0.496

**Table:** (**C**) Trained CPT for Right Stride Length

RSL${}_{T-1}$		Normal			Abnormal	
**CAD**	**Fast**	**Slow**	**Normal**	**Fast**	**Slow**	**Normal**
Normal	0.665	0.310	0.707	0.314	0.351	0.424
Abnormal	0.335	0.670	0.293	0.686	0.649	0.576

**Table:** (**D**) Trained CPT for Left Ankle Joint Angle

LSL	Normal	Abnormal
Normal	0.777	0.568
Abnormal	0.223	0.432

**Table:** (**E**) Trained CPT for Left Knee Joint Angle

LSL	Normal	Abnormal
Normal	0.161	0.088
Abnormal	0.839	0.912

**Table:** (**F**) Trained CPT for Right Ankle Joint Angle

RSL	Normal	Abnormal
Normal	0.723	0.578
Abnormal	0.277	0.422

**Table:** (**G**) Trained CPT for Right Knee Joint Angle

RSL	Normal	Abnormal
Normal	0.115	0.098
Abnormal	0.885	0.902

**Table 7:** Average value of correct classification rate, sensitivity and specificity ± standard deviation for Dataset A.

Classifier (Dataset A)	CCR	Sensitivity	Specificity
Linear Discriminant Analysis (LDA)	64.52 ± 1.08	68.00 ± 2.63	44.44 ± 2.54
Naive Bayes (NB)	77.62 ± 1.08	74.81 ± 1.21	66.67 ± 1.32
KNN (10 Neighbors)	70.24 ± 0.73	65.15 ± 1.85	87.25 ± 2.31
SVM	65.28 ± 0.61	65.63 ± 1.11	12.50 ± 0.80
Proposed Method (DBN)	86.9 ± 0.23	80.36 ± 1.28	90.18 ± 1.61

**Table 8:** Average value of correct classification rate, sensitivity and specificity ± standard deviation for Dataset B.

Classifier (Dataset B)	CCR	Sensitivity	Specificity
Linear Discriminant Analysis (LDA)	82.04 ± 1.09	91.18 ± 2.85	79.59 ± 2.13
Naive Bayes (NB)	80.77 ± 0.76	83.54 ± 1.63	80.34 ± 1.54
KNN (10 Neighbors)	83.45 ± 0.47	82.17 ± 1.95	85.54 ± 2.16
SVM	84.60 ± 0.14	94.67 ± 0.89	82.77 ± 0.78
Proposed Method (DBN)	88.68 ± 0.23	86.89 ± 1.12	91.96 ± 1.35

**Table 9:** Comparison with other model based methods.

Methods	Accuracy	# of Features Selected
KNN(10 Neighbors) [35]	88.54%	2
Logistic Regression (LR) [18]	89.2%	18
Naive Bayes (NB) [17]	94.1%	3
Proposed Method (DBN)	88.68%	7
